# Inducible nitric oxide synthase 2 promoter polymorphism and malaria disease severity in children in Southern Ghana

**DOI:** 10.1371/journal.pone.0202218

**Published:** 2018-08-17

**Authors:** Mawuli Dzodzomenyo, Anita Ghansah, Nana Ensaw, Benjamin Dovie, Langbong Bimi, Reginald Quansah, Ben A. Gyan, Mawuli Gyakobo, Benjamin Amoani

**Affiliations:** 1 Department of Biological, Environmental and Occupational Health Sciences, School of Public Health, University of Ghana, Legon, Ghana; 2 Noguchi Memorial Institute for Medical Research, University of Ghana, Legon, Ghana; 3 St. Dominic’s Hospital, Akwatia, Ghana; 4 Department of Geography, University of Ghana, Legon, Ghana; 5 Department of Animal Biology and Conservation Sciences, University of Ghana, Legon, Ghana; 6 Tetteh Quarshie Memorial Hospital, Mampong Akwapim, Ghana; 7 Department of Biomedical Sciences, College of Health and Allied Sciences, University of Cape Coast, Cape Coast, Ghana; Liverpool School of Tropical Medicine, UNITED KINGDOM

## Abstract

**Objective:**

We assessed the association of mutant allele frequencies of nitric oxide synthase 2 (NOS2) gene at two SNPs (-954 and -1173) with malaria disease severity in children from a malaria endemic area in Southern Ghana.

**Method:**

Using children recruited at the hospital, assigned into clinical subgroups of uncomplicated and severe malaria and matching with their “healthy control” counterparts, we designed a case control study. Genomic DNA was extracted and genotyping using Restriction Fragment Polymorphism was done.

**Result:**

A total of 123 malaria cases (91 uncomplicated, 32 severe) and 100 controls were sampled. Their corresponding mean Hbs were 9.6, 9.3 and 11.2g/dl and geometric mean parasite densities of 32097, 193252 and 0 parasites/ml respectively. Variant allele frequencies varied from 0.09 through 0.03 to 0.12 for G-954C and 0.06 through 0.03 to 0.07 for C-1173T in the uncomplicated, severe and healthy control groups respectively. There was a strong linkage disequilibrium between the two alleles (p<0.001). For the -954 position, the odds of developing severe malaria was found to be 2.5 times lower with the carriage of a C allele compared to those without severe malaria (χ^2^; p< 0.05) though this isn’t the case with -1173.

**Conclusion:**

The carriage of a mutant allele in the -954 NOS2 gene may have a protective effect on malaria among Southern Ghanaian children.

## Introduction

There is accumulating evidence that host genetic factors control and regulate the outcome of malaria disease by regulating immune responses and natural selection over many years may have led to variations in susceptibility in infected individuals. In malaria, much of the variations in disease susceptibility can be attributable to the protective roles of several genetic disorders of the red blood cell such as the sickle cell trait [1], the thalassaemias [2] and glucose-6-phosphate dehydrogenase deficiency [3].

Host resistance to malaria has been complex, and various associations of genetic polymorphisms with malaria severity have been postulated. Of recent times, the role of cytokines in controlling the intensity and duration of immune responses to malaria infection in different populations has been studied. Polymorphisms in genes encoding such cytokines as the tumor necrosis factor (TNF-α) and the increased risk of cerebral malaria has been documented [4, 5]. Genetic variants in the Th2 cytokine IL-4 are also known to result in severe malaria or confer some protection against clinical outcomes by mediating the levels of antibodies [4, 6, 7].

Nitric oxide (NO) is known to mediate host resistance to infectious organisms [8] and is produced by three different nitric oxide synthases in humans. Inducible nitric oxide synthase (NOS2) produces nitric oxide (NO), which is thought to be toxic to malaria parasites in vitro [9, 10] and also mediates host protective effects in rodent models of malaria [11]

There has been much speculation about the role of NO in malaria and recent reports identified associations between polymorphisms in the promoter region of the gene encoding NOS2 and malaria disease severity. To date, four polymorphisms in the NOS2 promoter have been associated with variable malarial disease outcomes. These include a CCTTT microsatellite repeat located 2.5 kilobases upstream from the NOS2 transcriptional start site [12], and three single nucleotide polymorphisms; -954 (G-954C) [13], -1173 (C-1173T) [14] and -1659 (A-1659G) [12]. Studies in different geographical locations have, however, documented conflicting views of the functional relevance of these polymorphisms. Both in vitro and in vivo studies reported conflicting results for the pentanucleotide microsatellite repeat CCTTT polymorphism. While shorter forms of the repeat are associated with cerebral malaria in a cohort of Gambian children [15], in Thai adults, severe forms of malaria correlated positively with longer forms of this variant [16]. In contrast, repeat length was not associated with malarial disease severity in studies in neither Tanzania [17] nor Gabon [18].

Though large-scale genomic studies are currently being advocated, the design of these studies does not capture the basic information from Africa and therefore may not be suitable in the African setting. Until these are designed to suit the African settings, a small study of this nature may produce useful information about genes and disease association, since we postulate that NOS2 promoter polymorphism could determine malaria disease severity. In addition, in Ghana, there are three ecological zones with different malaria endemicities and earlier studies looked at SNPs in the savannah belt. This study therefore presents the information about the correlation of NOS2 SNPs in the forest area of Ghana. In this study, using a case-control approach, we hypothesize that in a cohort of Southern Ghanaian children, mutant allele frequencies at two SNPs (-954 and -1173) of the inducible nitric oxide synthase 2 promoter gene correlates positively with malaria disease severity.

## Materials and methods

### Ethical statement

Approval and ethical clearance for the study was obtained from the Institutional Review Board of the Noguchi Memorial Institute for Medical Research (NMIMR), University of Ghana, Legon. (No. IRB 0001276). An information session was presented to the study participants prior to obtaining written consent. Adolescents and children provided assent in addition to the required informed consent of a parent or guardian.

### Study design and area

The study design was an age and sex-matched case-control study. The study was conducted at the St. Dominic District Hospital at Akwatia in the Eastern Region of Ghana. Study participants were children presenting at the hospital with confirmed malaria after an initial investigation. These children who were designated as cases were matched with their “healthy control” counterparts, who were children in the same age group, recruited from the community. All children who tested positive for sickling (including all other haemoglobinopathies) and helminth infections were excluded. Aside cerebral malaria, other causes of encephalopathy including viral encephalitis, uraemia, and hypertensive encephalopathy from glomerulonephritis and head injury from trauma were also excluded. A finger prick blood was taken from both categories of children for the preparation of thick and thin films for microscopy to diagnose malaria and blood was also collected into heparinised capillary tubes (Drummond Scientific Company, USA) and blotted on chromatographic filter paper (ET31CHR, Whatman Limited, England) for DNA extraction and subsequent molecular analysis.

### Study subjects

For severe malaria, the inclusion criteria was children with asexual *P*. *falciparum* parasitaemia, with at least one of the following signs; prostration, unrousable coma (score between 0 and 2 on the Blantrye coma scale [19]. These subjects were also children with asexual *P*. *falciparum* parasitaemia with haemoglobin levels < 5g/dl and no other cause of anaemia. Uncomplicated malaria cases were children with clinical illness characterized by an axillary temperature >37.5°C associated with *P*. *falciparum*–positive blood smear and haemoglobin level of > 8g/dl. Control subjects were children recruited from the surrounding communities where the cases came from and were children who were “healthy” and therefore aparasitaemic. They were recruited from homes, crèches and kindergartens and were age-matched with their case counterparts.

### DNA extraction, PCR and genotyping

DNA was extracted from 5-6mm of filter paper after punching using the Tris-EDTA method. Polymorphisms were detected using nested polymerase chain reaction (nPCR) as described previously [20]. This involved amplifying a 680 bp fragment of the NOS2 gene and for G954C using a first round PCR with primers 5’-CGGCATCTTGTCACTTTCTAA-3’ and 5’-GGGAGATTTTTTCCTCAGC-3 under the following conditions; 94°C for 5 min, amplification for 25 cycles at 94°C for 1 min, 52°C for 2 min, 72°C for 2 min and finally 72°C min for 8 min. For each reaction, a negative control which contained no DNA but sddH_2_O was added in the amplification. For the second round, the primers 5’-CATATGTATGGGAATACTGTATTTCAGGC-3’ and 5’TCTGAACTAGTCACTTGAGG-3’ were used under the following conditions; 92°C for 3min, followed by 40 cycles at 92°C for 30s, 60°C for 1 min, 72 °C for 1 min and final cycle at 72°C for 7 min. The second round conditions for C1173T involved using the primers 5’-CTCCATAAGCCAGAGCTCTAA-3’ and 5’-CCACCACACCCAGCTAATATTT-3’ with cycling conditions as; 92°C for 3min followed by 40 cycles at 92°C for 30s, 56°C for 1 min, 72°C for 1 min and final cycle at 72°C for 7 min. To determine polymorphism in G954C, the fragments generated were subjected to digestion with restriction endonucleases *Bsa*I (New England BioLabs, Beverly, MA). For C1173T, fragments were digested with *Fok*I (New England BioLabs, Beverly, MA). The *Bsa*l and *Fok*I-digested PCR products were analyzed on 2% agarose gel.

### Statistical analysis

Allelic frequency distribution was tested according to the Hardy-Weinberg equilibrium. The χ^2^ test was also used to estimate the association between the polymorphisms and the different clinical sub-groups. The SPSS ver. 17 statistical package was used for statistical analysis and p values <0.05 were considered significant.

## Results

### Characteristics of the major clinical subgroups and their control counterparts

Table 1 shows the characteristics of the major clinical subgroups and their control counterparts. Majority of the cases had uncomplicated malaria (71%), while severe malaria, in this case defined as fever with parasite density >100,000, in addition to one or more of the other severe malaria symptoms were 39 (29%). The mean Hb for the severe malaria cases were slightly lower (9.3g/dl) than that for the uncomplicated malaria group whose mean Hb was 9.6. The mean Hb for the controls however was 11.2 g/dl. The Geometric Mean Parasite Density was 32097.2 and 193252.4 for the uncomplicated and severe malaria cases respectively and was statistically significant (χ^2^, p<0.05). There was also significant difference (χ^2^, p<0.001) in the mean temperatures for the case and control group. Mean temperatures were highest in the severe malaria cases (38.2) than in the uncomplicated cases and the healthy controls which were 37.8 and 36.7 °C respectively. *Plasmodium falciparum* was the predominant parasite species in both clinical groups (uncomplicated and severe malaria). Four children who had uncomplicated malaria were harbouring *Plasmodium malariae* while mixed infection of both species was found in five children.

**Table 1:** Characteristics of the major clinical subgroups and their healthy control counterparts.

Variables		Uncomplicated malaria	Severe malaria	Healthy controls	P- value
N		91	32	100	
Gender	(Male)	51	17	55	
	(Female)	40	15	45	
Mean Age (Months)		34.3	31.0	43.68	
Mean Hb (g/dl)		9.6 (7.86,11.40)	9.3 (8.80,9.85)	11.2 (11.02,11.36)	0.001
*GMPD (parasites/ml)		32097	193252	-	0.045
Mean Temp (°C)		37.8 (37.58, 37.99)	38.2 (37.78,38.56)	36.7 (36.46,37.02)	0.001
Parasite species					
*P*.*f*		85	29		
*P*.*m*		4	0		
*P*.*f/P*.*m*		2	3		0.001

*GMPD = Geometric Mean Parasite Density, N = number, *P*.*f* = *Plasmodium falciparum*,

*P*.*m = Plasmodium malariae*, *P*.*f/P*.*m* = Co-infection

### Mutant allele frequencies of the NOS2 gene

Table 2 summarises the distribution of genotypes and allele frequencies of the two NOS2 polymorphic positions in the study children. A total of 223 samples were typed for the two positions of NOS2-954 and -1173. For NOS2-954, C allele frequencies were 0.09, 0.03 and 0.12 for uncomplicated, severe malaria and the healthy control group respectively and for NOS2-1173, the frequencies were 0.06, 0.03 and 0.07 for the same clinical groupings.

**Table 2:** Mutant allele frequencies in the two positions of the iNOS2 gene in the different clinical groups.

Group	NOS2–954					NOS2-1173			
	n	GG	GC	CC	C allele frequency	CC	CT	TT	T allele frequency
UM	91	74(81.6)	16(17.6)	1(1.1)	0.09	79(86.6)	12(13.2)	0	0.06
SM	32	30(93.7)	2(6.3)	0	0.03	30(93.7)	2(6.3)	0	0.03
HC	100	79 (79)	19(19)	2(2)	0.12	86(86)	14(14)	0	0.07
Total	223	183(82)	37(17)	3 (0.03)	0.09	195(87)	28(13)	0	0.07

UM = uncomplicated malaria; SM = severe malaria; HC = healthy control

### Association of mutant allele frequencies and malaria disease severity

Table 3 shows the analysis of the association of the polymorphisms in the different clinical groups. For the NOS2-954 position, the odds of developing severe malaria was found to be 2.5 times lower in those with the carriage of a C allele compared to those without severe malaria (OR: 0.25; CI 0.03–1.06; p<0.05). The association of this polymorphism with uncomplicated malaria however was not statistically significant. The association between the mutant alleles of NOS2-1173 and disease severity however did not show any significance.

**Table 3:** Association between genotypes and malaria disease severity in children.

	Genotype	SM	UM	HC			
		(32)	(91)	(100)	SM vs. HC	UM vs. HC	SM vs. UM
SNP	GG	30(93.7)	74 (81.3)	79 (79)	Reference	Reference	reference
-954G →C	GC	2 (6.3)	16 (17.6)	19 (19)	0.28, (0.06–1.27), 0.057	0.9, (0.43–1.88), 0.77	0.31, (0.067–1.42), 0.114
	CC	0 (0)	1 (1.1)	2 (2)	-	0.53, (0.05–5.99), 0.6	-
	Alleles						
	G	62(0.97)	164(0.901)	177(0.885)	Reference	Reference	Reference
	C	2 (0.03)	18(0.099)	23(0.115)	0.25, (0.03–1.06), 0.046	0.84, (0.41–1.70), 0.611	0.29, (0.03–1.29), 0.088
-1173C →T	CC	30(93.7)	79 (86.8)	86 (86)	Reference	Reference	reference
	CT	2 (6.3)	12 (13.2)	14 (14)	0.41, (0.09–1.92), 0.21	0.93, (0.41–2.14), 0.87	0.44, (0.045–2.17), 0.29
	TT	0	0	0	-	-	-
	Alleles						
	C	62(0.97)	170 (0.934)	186 (0.93)	Reference	Reference	reference
	T	2 (0.03)	12(0.066)	14 (0.07)	0.43, (0.14–1.38), 0.46	0.94, (0.42–2.09), 0.86	0.44, (0.045–2.17), 0.29

UM = uncomplicated malaria; SM = severe malaria; HC = healthy control; SNP = single nucleotide polymorphism.

## Discussion

In this study, our aim was to use a case-control approach to determine the association of genetic polymorphisms in the NOS2 gene and its relation with malaria disease severity in children in Southern Ghana. This is also the first study in Southern Ghana to document how malaria outcomes are affected by genetic polymorphisms in an endemic population. We found that the G954C polymorphism was present in the Southern Ghanaian population with an allele frequency of 9%. This is consistent with other findings from sub-Saharan African malaria-endemic populations, which varied from 7% to 15% [17, 18] compared with only 1–4% in low malaria-endemic populations. The C1173T polymorphism however was present in the study population with an allele frequency of 7%, which is slightly higher than the 4% allele frequency recorded in Tanzanian adults and Western Kenyan children [15]

We found evidence for a protective effect of the G954C SNP against severe malaria in this cohort of Ghanaian children. This contrasts earlier findings from Northern Ghana where no evidence was found for the protective effect of the G954C SNP against severe malaria [19], and in Gambia, East Africa and India [12–14, 21]; where the G954C SNP was identified to be associated with malaria disease severity. Our result was however, consistent with findings from Gabon, Uganda, Tanzania and Kenya [15, 18, 22–24]; where the G954C SNP was found to have a protective effect against severe malaria. Other studies in Tanzania and Gabon found no association between G954C and cerebral malaria or the incidence of uncomplicated malaria [15], although the study did not include younger children most likely to be protected by higher levels of nitric oxide. However, we found no clear evidence for C1173T SNP protection against severe malaria which is in accordance with the previous observations made by other investigators in Northern Ghana [19] and Uganda [20]. This contrasts the findings by Levesque et al. [15], in Tanzania, where the C1173T SNP was found to reduce the risk of symptomatic malaria by almost 90%, and that of severe malarial anaemia in Kenya by 69%, and Kumar et al. [21], in India, where NOS2 1173CC+CT genotypes and the NOS-1173 TT genotypes were observed to be associated with an increased risk of malaria.

The noted differences in NOS2 promoter polymorphisms and disease severity between our population of Southern Ghanaian (West African) children and the populations of Gabonese (Central African) and Tanzanian (East African) children may indicate differences in the genetic backgrounds of each population, differences in the strains of malaria, or differences in the epidemiology of malaria infection in these regions. The functional significance of these conflicting observations is ambiguous, suggesting that the relationship between NOS2 polymorphism and malaria severity is much more complex [15, 21], Thus, the role of NOS2 polymorphisms may vary with endemic regions across the globe as does the manifestation of malaria [21]

Moreover, our findings are pertinent and their interpretation can be attributed to several reasons because the NOS2-954 polymorphism is located in the NOS2 promoter region, which could be an activator of gene expression and nitric oxide production [13]. The heterozygote G954C allele of NOS2 gene may have a higher affinity to a DNA binding protein, but not the wild type of NOS2-954G allele [21]. Hence, the translational activity and NOS mRNA levels may have increased in the iNOS-954C allele resulting in overall enhanced NOS expression and its accomplished protection against severe malaria in our study cohort. This single nucleotide polymorphism has been shown to modify NOS2 transcription and increase nitric oxide activity that may be important in parasite clearance [18, 19] and protection against *P*. *falciparum* infection [25–26]. Genetic variation in NOS2 promoter region may play a very important biological role in host defense mechanisms against malaria pathology by enhancing anti-oxidants enzymes and stimulating the infection induced biochemical cascade against plasmodium pathology [21].

Among other functions, nitric oxide appears to be protective in endothelial function including reducing blood cell-endothelial cell adhesion in studies both in humans and animal models and ameliorating deleterious cytokine production [27]. Compared to individuals with severe forms of malaria, higher levels of nitric oxide metabolites have been associated with mild malaria in Tanzanian children and Indonesian adults [10, 28]. Differences in the genes encoding for NOS2 which may result in different nitric oxide productions between uncomplicated forms and their severe counterparts may be the differences between these clinical groups. However, an obvious limitation in our study is that effects of innate immune system on infectious disease are multifactorial, and NO production is just one of many factors that have been recognised as influencing susceptibility to and severity of infections. In conclusion, our study shows that the carriage of a mutant allele in the -954 NOS2 gene may have a protective effect on malaria among Southern Ghanaian children.

## Supporting information

S1 Table The demographic and parasitological study data obtained from the study participants (NOS2 Project data for test samples.xlsx). (XLSX)

S2 Table The demographic and molecular study data obtained from the study participants (NOS2 result for PCR analysis (control and cases samples).xlsx). (XLSX)
